# Supplementary material for: Transient risk of ambient fine particulate matter on hourly cardiovascular events in Tainan City, Taiwan
Source: PLoS One. 2020 Aug 21;15(8):e0238082. doi: 10.1371/journal.pone.0238082 (PMC7442245; doi:10.1371/journal.pone.0238082)
Supplement: Attachment — Submitted filename: Response to Reviewers Cover letter PLos One.doc [file pone.0238082.s002.doc]

August. 1, 2020

Dear Editor/Reviewers:

Re: PONE-D-20-18170

My colleagues and I would like to resubmit this revised manuscript entitled “Transient Risk of Ambient Fine Particulate Matter on Hourly Cardiovascular Events in Tainan City, Taiwan” for your consideration of publication as a Research Article for the special issue of “[*Primary and Secondary Prevention of Cardiovascular Disease*](https://collections.plos.org/s/prevention-cardiovascular)” in the *“PLos One”.*

We thank for editor and reviewers’ comments. We revise manuscript addressing the response to editor and reviewer's original comment(s). The point-by-point response to editor and reviewer lists as below:

<<Response to Journal Requirements>>

1. Please provide additional details regarding participant consent. In the ethics statement in the Methods and online submission information, please ensure that you have specified (1) whether consent was informed and (2) what type you obtained (for instance, written or verbal, and if verbal, how it was documented and witnessed). If your study included minors, state whether you obtained consent from parents or guardians. If the need for consent was waived by the ethics committee, please include this information.

Response: We added the statement about the waiving inform consent approval information “This study is approved by the Institutional Review Board of Chi Mei Medical Center on 14^th^ Dec. 2018 (No.10612-012) and is exempt from obtaining inform consent.” in line 122-4.

<<Response to Reviewer #1>>

1. Reviewer #1: I found some merits in this methodology, the results obtained are interesting and deserve to be disclosed, as they could provide useful information to policymakers. The topic exposed is in compliance with the arguments required in this journal and the format is respected too. The statistical analysis performed is good and I find the application in this field original. However, I suggest improving the environmental aspect of air quality in big cities adds some specific works to make this work more complete. I suggest you some papers about it:

-Row 302 add: “Analysis of vertical profile of particulates dispersion in function of the aerodynamic diameter at acongested road in Catania”

-Row 318 add: “Evaluation of the air pollution in a Mediterranean region by the air quality index”

-Row 326 add: “Air quality data for Catania: analysis and investigation case study 2012-2013”

Response: Thanks for reviewer’s comments and suggestions. We add a sentence “Studies conducted in urban building environment also demonstrate vertical and horizontal differences[32, 33].” at row 303-304 and cite more articles [36, 37] at row 320 to enrich our manuscript.

This letter will serve to certify that we have fully response all the reviewers’ comments and journal requirements. We hope for the reviewers’ and editor’s consideration in publishing this manuscript and look forward to hearing from you soon.

Sincerely,

Tain-Junn Cheng, MD, PhD
